# Supplementary material for: Drug repurposing for aging research using model organisms
Source: Aging Cell. 2017 Jun 16;16(5):1006–15. doi: 10.1111/acel.12626 (PMC5595691; doi:10.1111/acel.12626)
Supplement: Supplementary file 7 — Data S1 Zip‐Archive of all report cards. [file ACEL-16-1006-s007.zip › RC_EKZ.pdf]

# EKZ

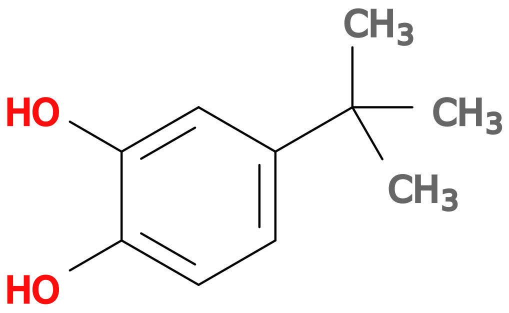


**Database identifiers**

| | |
|---|---|
| ChEMBLCompound | CHEMBL220845 |
| ZINC | ZINC00388150 |
| eMolecules | 475721 |

## Ranking

| | Rank | Score |
|---|---|---|
| Drosophila | 506/697 | 0.265 |
| C. elegans | NA | NA |

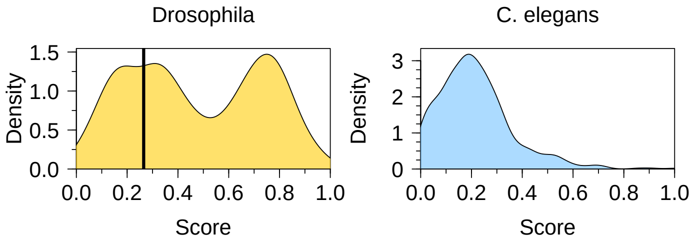


| | Ageing implication | Domain conservation | Binding site conservation | Binding affinity | Bioavailability | Lipinski | Promiscuity | Purchasability | Drug approval | **Total** |
|---|---|---|---|---|---|---|---|---|---|---|
| Drosophila | 1.0 | 0.917 | 0.691 | 0.29 | (0.9) | 0.0 | -0.0 | 0.1 | 0.0 | 0.265 |
| C. elegans | NA | NA | NA | NA | NA | NA | NA | NA | NA | NA |

## Names

No synonyms found

## Roles

ChEBI entry None has no roles

## Status

| | |
|---|---|
| Approved drug (according to ChEMBL) | No |
| Number of Rule of 5 violations | 0 |
| Binding affinity to original target in log units (RF-Score prediction) | 4.11 |
| Burns *C. elegans* bioavailability prediction | 1.63 |

## Compound Target Characteristics

### Peroxiredoxin-5, mitochondrial

Best gene implication in ageing for this target family came from gene Q960M4 annotated in UniProt release 2014_02. Annotation GO 8340 (determination of adult lifespan) was Inferred from Mutant Phenotype

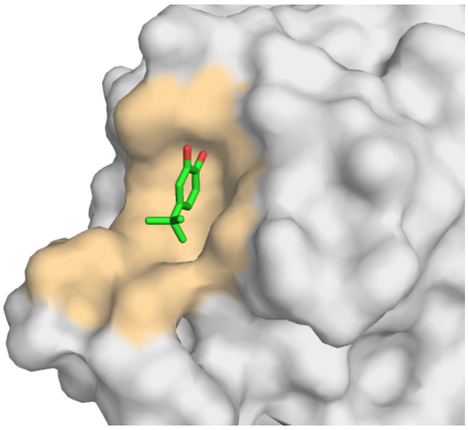

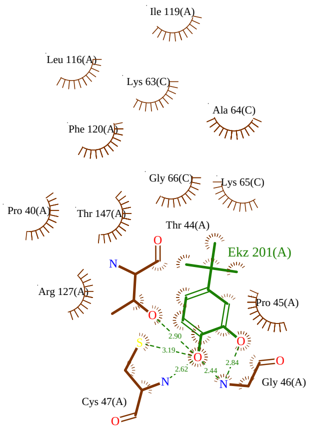


| protein | amino acids contacts (binding site) |
|---|---|
| PDB:4k7o:chainA:P30044 | P T P G C L I F R T |
| sp:P30044:PRDX5_HUMAN | P T P G C L I F R T |
| tr:D3ZEN5:D3ZEN5_RAT | P T P G C L L F R T |
| sp:Q9R063:PRDX5_RAT | P T P G C L L F R T |
| tr:G3UZJ4:G3UZJ4_MOUSE | P T P G C L L F R T |
| tr:Q3UWS9:Q3UWS9_MOUSE | P T P G C L L F R T |
| sp:P99029:PRDX5_MOUSE | P T P G C L L F R T |
| tr:H3BJQ7:H3BJQ7_MOUSE | P T P G C L L F R T |
| tr:Q960M4:Q960M4_DROME | P T P G C - P L R T |
| sp:P38013:AHP1_YEAST | P S P T C L G D R T |

| | whole protein | | domain-based | | contact-based | |
|---|---|---|---|---|---|---|
| protein | ident | simil | ident | simil | ident | simil |
| PDB:4k7o:chainA:P30044 | 0.99 | 1.0 | 0.99 | 1.0 | 1.0 | 1.0 |
| sp:P30044:PRDX5_HUMAN | 1.0 | 1.0 | 1.0 | 1.0 | 1.0 | 1.0 |
| tr:D3ZEN5:D3ZEN5_RAT | 0.66 | 0.73 | 0.84 | 0.93 | 0.9 | 1.0 |
| sp:Q9R063:PRDX5_RAT | 0.78 | 0.91 | 0.89 | 0.97 | 0.9 | 1.0 |
| tr:G3UZJ4:G3UZJ4_MOUSE | 0.59 | 0.7 | 0.66 | 0.72 | 0.9 | 1.0 |
| tr:Q3UWS9:Q3UWS9_MOUSE | 0.76 | 0.87 | 0.89 | 0.97 | 0.9 | 1.0 |
| sp:P99029:PRDX5_MOUSE | 0.77 | 0.9 | 0.89 | 0.97 | 0.9 | 1.0 |
| tr:H3BJQ7:H3BJQ7_MOUSE | 0.66 | 0.78 | 0.85 | 0.95 | 0.9 | 1.0 |
| tr:Q960M4:Q960M4_DROME | 0.46 | 0.73 | 0.57 | 0.84 | 0.7 | 0.69 |
| sp:P38013:AHP1_YEAST | 0.19 | 0.55 | 0.23 | 0.64 | 0.6 | 0.6 |

### Prx5 (FBgn0038570) associated phenotypes

chemical resistant, chemical sensitive, immune response defective, increased cell death, long lived, parental effect, short lived, stress response defective

(Information from FlyBase)
